# Obesity related to metabolic syndrome: comparison of obesity indicators in an older french population

**DOI:** 10.1186/s13098-023-01078-x

**Published:** 2023-05-11

**Authors:** Hourfil-Gabin Ntougou Assoumou, Vincent Pichot, Jean-Claude Barthelemy, Sébastien Celle, Arnauld Garcin, Thierry Thomas, Frédéric Roche

**Affiliations:** 1 Ecole Normale Supérieure, Libreville, Gabon; 2 Clinical and Exercise Physiology Laboratory, University Hospital, Saint-Etienne, F-42055 France; 3 INSERM, U1059 Sainbiose, Jean Monnet University, Mines Saint Etienne Faculté de Médecine, Campus Santé Innovation, Saint-Étienne, F-42023 France; 4 Rhumatology Dpt, University Hospital, Saint-Etienne, F-42055 France; 5 Centre VISAS, Bâtiment A, CHU Nord, Saint Etienne Cedex 2, F-42055 France

**Keywords:** Metabolic syndrome, Body fat index, Body mass index, Obesity, Abdominal obesity, Waist circumference, Adiposity

## Abstract

**Objectives:**

Metabolic syndrome (MS) represents a cluster of metabolic abnormalities. Insulin resistance is a major component of the syndrome. We analyze in this study the relationship between body fat composition and MS in comparison to usual obesity indicators in an older adult population.

**Design:**

: The PROgnostic indicator OF cardiovascular and cerebrovascular events (PROOF) study is a prospective longitudinal community cohort study among the inhabitants of Saint-Etienne, France.

**Methods:**

The study is a cohort study of 1011 subjects, mean age 65.6 ± 0.8 years old at inclusion, recruited from the electoral list of the town in 2000. Among them, 806 subjects realized a Dual-energy X-ray absorptiometry (DXA) used to evaluate body fat and lean mass repartition. We evaluate biological metabolic parameters according to usual techniques. The indices of obesity were calculated according to standard formula. MS presence and its components were simultaneously evaluated.

**Results:**

All obesity parameters were significantly higher (p < 0.0001) in subjects suffering metabolic syndrome as compared to those without. Body fat index (BFI) presented a stronger correlation to total fat mass, trunk fat mass and body adiposity index (BAI). The correlations between body indices and metabolic components showed that body mass index (BMI) and waist circumference were more strongly associated with BFI as compared to BAI and total fat mass. According to logistic regression analysis, only the waist-hip ratio (WHR) demonstrated a significant association with MS severity (p < 0.0001).

**Conclusions:**

Among the obesity indices, BFI and BAI represented the best indicators to characterize global obesity while WHR only is highly predictive of metabolic syndrome presence and severity. The BAI indicator is an alternative for measuring obesity. Comparison of long-term impact of such markers on cardiovascular morbidity and mortality is now questioned.

## Introduction

The metabolic syndrome (MS) is a group of metabolic abnormalities with the insulin resistance as a major component. It has gone by several different names over the past two decades. The diagnostic criteria proposed by the Adult Treatment Program III (ATP III) of the National Cholesterol Education Program have led to a better understanding of the components and treatment strategies. Five diagnostic criteria have been listed in the ATP III version of MS, [1]. The presence of three of these five criteria is considered necessary and sufficient to assess the diagnosis.

The prevalence of metabolic syndrome is increasing worldwide, especially in the elderly. Elderly subjects, due to the impact of aging, are at increased risk of developing “android”, abdominal obesity and associated metabolic syndrome. Abdominal adiposity [2] has been associated with a high risk of cardiovascular events [3]. Early studies focused on the waist-to-hip circumference (WHC) ratio as a key measure. More recent studies have shown that an elevated waist circumference (WC) is an independent factor for increased risk of cardiovascular events, hyperinsulinemia, and increased insulin resistance in metabolic studies. This high waist circumference is also correlated with an increase in abdominal fat assessed by techniques such as CT scans [4]. Fat deposits can be subcutaneous and/or intra-abdominal with very different cardiovascular consequences. Waist circumference (WC) and waist-to-hip ratio (WHR) are therefore often used in epidemiological and clinical settings as a simple means of quantifying the distribution of body fat and thus sometimes allow the diagnosis of central adiposity. However, considerable amounts of fat can also be present within the muscles, particularly in the elderly (sometimes associated with true sarcopenia). It is well recognized that adipose tissue has different characteristics depending on its anatomical site of development. Subcutaneous fat and lean body mass (appendicular) seem to have opposite associations with metabolic disorders and cardiovascular risk factors. This may be because subdividing body content into lean mass and fat mass, and body fat into subcutaneous and visceral fat, is at the same time approximate and inappropriate for studies testing the metabolic consequences of these disorders. A multi-compartmental analysis of the human body at the tissue (and organ) level could be much more accurate and would help to better understand the complex interrelationships between human body composition and metabolism [5].

While new indicators of obesity characteristics such as the body fat index (BFI) and body adiposity index (BAI) have been proposed in epidemiology, they are still poorly developed in clinical use. Moreover, their association with the metabolic syndrome is still poorly established.

The aim of this study is to analyze the relationship between body fat composition and metabolic syndrome in relation to these indicators of obesity in a 65-year-old population included in the PROOF cohort study (Loire, France).

## Materials and methods

### Subjects

The PROgnostic indicator OF cardiovascular and cerebrovascular events study, the PROOF study (NCT:00759304) [6] was designed to assess the predictive capacity of features depicting the level of ANS activity level regarding cardiovascular events and mortality. In 2001 and 2002, an invitation to participate to the PROOF study was exhaustively sent to every people aged 65 living in Saint-Étienne, France. A prospective cohort study was then performed from the 1011 subjects (mean age 65.6 ± 0.8 years old) who answered positively. Subjects with prior myocardial infarction, stroke, congestive heart disease, type 1 diabetes, and dependant people or people living in institutions were excluded from the study. The PROOF study was approved by the IRB-IEC (CCPRB Rhone-Alpes Loire). All participants signed a consent letter prior to the study.

### Assessment of metabolic risk factors

During the clinical evaluation, the height in stocking feet and weight in light clothing were measured and the body mass index (BMI) was calculated as weight (kg)/height squared (kg/m^2^). WHR (waist to hip ratio) was calculated as waist circumference / hip circumference according to the standard. Waist was measured midway between the lower rib margin and the iliac crest and Hip at the widest part of the buttock. For stratified analyses, overweight was defined according to the National Institutes of Health clinical guidelines for adults as a BMI > 25 kg/m^2^ and obesity as a BMI > 30 kg/m^2^. BAI was calculated as [(hip circumference)/(height)^1.5^)] -18 [9].

Systolic (SBP) and diastolic (DBP) blood pressures were measured by a physician (FR/JCB) using a standard mercury sphygmomanometer on the right arm while the subject was quietly seated after at least 5 min of rest. Ambulatory blood pressure monitoring (Diasys Integra, Novacor, Rueil-Malmaison, France) was performed on a 24-hour period using the auscultatory method with a measure each 20 min during the day period and each 30 min during the night period. Blood for assessments of biological factors was taken in the morning after an overnight fasting. Serum levels of lipids, including total cholesterol, high density lipoprotein cholesterol (HDL), calculated low density lipoprotein cholesterol (LDL) from the Friedewald Formula, and triglycerides were assessed with flex reagent cartridge (Roche Diagnostic SA).

### Definition of the metabolic syndrome

Metabolic syndrome was defined at the inclusion according to the National Cholesterol Education Program criteria [7] as having three or more of the following metabolic risk factors: WC > 102 cm for men and > 88 cm for women, fasting plasma glucose concentration level > 6.1 mmol/l (110 mg/dl) or on medication, SBP > 130 mmHg or DBP > 85 mmHg or on medication, HDL concentration < 1.03 mmol/l (40 mg/dl) if male and < 1.29 mmol/l (50 mg/dl) if female, and fasting or non fasting triglyceride concentration > 1.69 mmol/l (150 mg/dl).

### Body composition

Dual-energy X-ray absorptiometry (DXA, Delphi WS/N70453) was used to evaluate body fat and lean mass composition in 806 subjects consecutively included in the PROOF study. DXA is a well accepted method for evaluating body composition and is now a standard because of its precision and simplicity. BFI was calculated as total body fat mass evaluated by DXA divided by the squared height in meters [8].

### Statistical analysis

Normally distributed data were presented as means and standard deviation of the means and categorical data in percentage. ANOVA with Fischer’s exact post hoc test was used to compare anthropometric and bioclinical data between subjects with and without metabolic syndrome. Multiple logistic regressions were performed to analyze the relationship between metabolic syndrome criteria and the different obesity indices. Stepwise logistic regression was performed to analyze the interrelations between the same parameters. A p-value < 0.05 was considered to be statistically significant. Data were analyzed using Statview 5 (SAS Institute) statistical software.

## Results

Our data showed that the increase of most anthropometric parameters was significantly linked (p < 0.0001) to metabolic syndrome. However, parameters such as fat head-neck composition did not significantly differ between groups **(**Table 1**)**. There was a high correlation among all body fat parameters at p < 0.0001 **(**Table 2**).**

**Table 1:** Anthropometric characteristics according to diagnosis of metabolic syndrome

	(Men = 344, 41.35%; Women = 488, 58.65%)		
Parameters	Metabolic syndrome – (n = 727; 90.20%)	Metabolic syndrome + (n = 79; 9.80%)	*P-value*
	Mean ± SD		
Weight (kg) Heigh (m) BMI (kg/m^2^)	67.62 ± 11.66 164.240 ± 8.36 25.00 ± 3.49	81.22 ± 10.73 165.456 ± 8.24 29.70 ± 3.62	< 0.0001 ns < 0.0001
BAI	28.40 ± 4.56	31.83 ± 6.80	< 0.0001
BFI (kg/m^2^)	8.01 ± 2.89	10.72 ± 3.51	< 0.0001
Fat mass %	31.75 ± 9.07	36.07 ± 8.87	< 0.0001
Fat trunk %	16.01 ± 4.57	18.82 ± 3.90	< 0.0001
Fat head %	1.89 ± 0.49	1.79 ± 0.39	ns
Glycaemia (g/L)	0.97 ± 0.05	1.21 ± 0.04	< 0.0001
LDL Chol (g/L)	1.36 ± 0.01	1.35 ± 0.04	ns
HDL Chol (g/L)	0.65 ± 0.05	0.48 ± 0.02	< 0.0001
Triglycerides (g/L)	1.00 ± 0.17	1.70 ± 0.09	< 0.0001
SBP (mmHg)	118.09 ± 0.50	127.52 ± 1.93	< 0.001
DBP (mmHg)	74.48 ± 4.28	76.6 ± 2.93	< 0.01

*Data are presented as mean ± standard deviation (SD)*

BFI: body fat index; BAI: body adiposity index; BMI: body mass index, Chol: cholesterol; SBP: mean 24 h systolic blood pressure; DBP: mean 24 h diastolic blood pressure

**Table 2:** Correlation coefficients matrix according to obesity indices

	BAI	BFI (kg/m^2^)	WC (cm)	BMI (kg/m^2^)	% total fat mass	% trunk fat mass
BAI	1					
BFI (kg/m^2^)	0.73***	1				
WC (cm)	0.34***	0.41***	1			
BMI (kg/m^2^)	0.51***	0.59***	0.65***	1		
% total fat mass	0.61**	0.91***	0.14***	0.29***	1	
% trunk fat mass	0.50***	0.81***	0.38***	0.37***	0.83***	1

BAI: body adiposity index; BFI: body fat index; WC: waist circumference; BMI: body mass index ***p < 0.0001

**Fig. 1:**
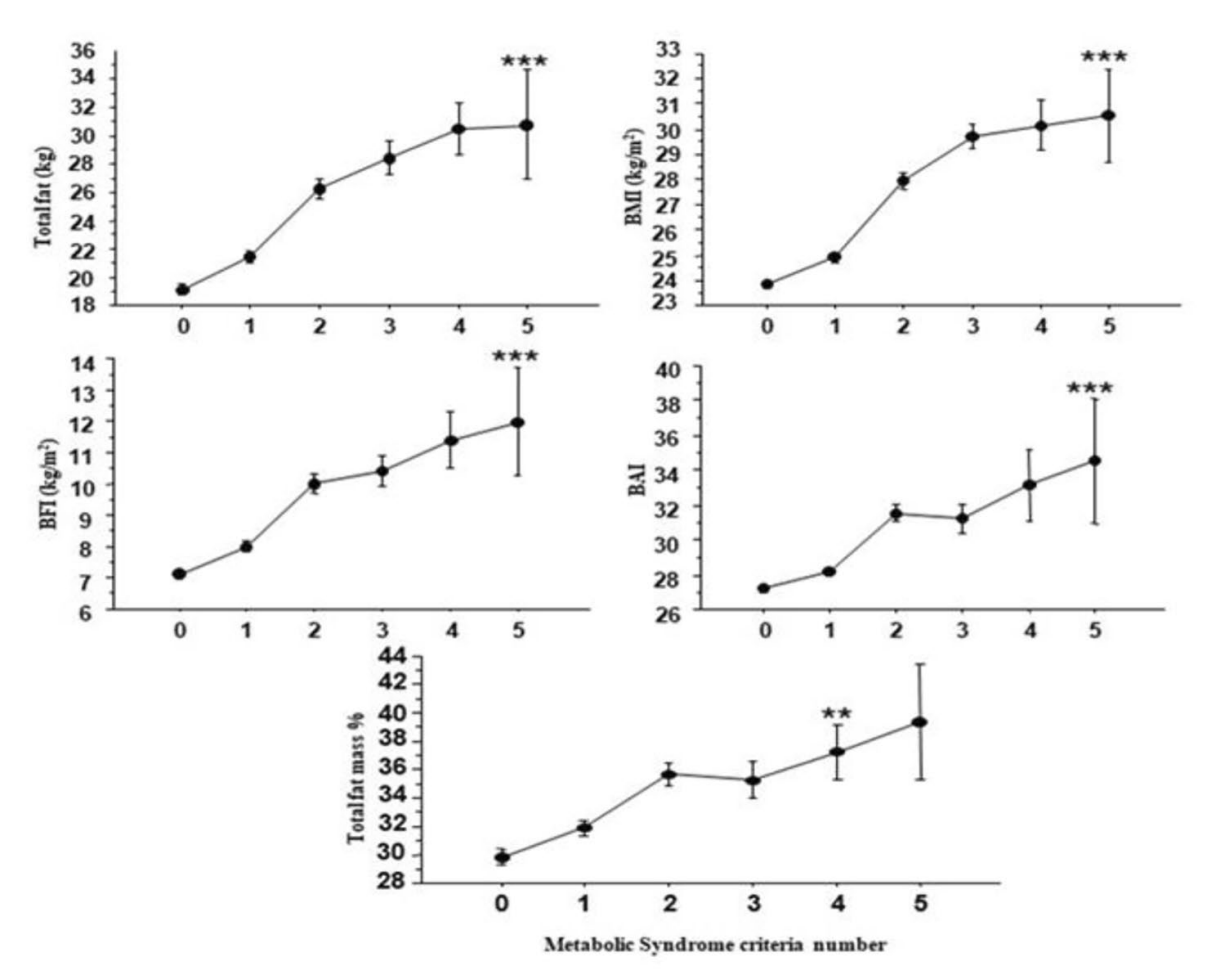
Evolution of fat mass indices according to metabolic syndrome criteria number. BAI: body adiposity index, BMI: body mass index, BFI: body fat index ** p < 0.001 ; ***p < 0.0001 (0 vs. 5)

The correlations between body indices showed that BMI, waist circumference, and trunk fat were more significantly associated with biological and clinical than the other indices **(**Table 3**).** From the logistic regression analysis, only WHR revealed a significant association with the five metabolic syndrome criteria (p < 0.0001) **(**Table 4**).** Finally, the indices Total fat mass, BFI, BAI, and BMI, were significantly altered only when five criteria of MS were present, excepted for Total fat mass % where the presence of 4 criteria were already associated with that alteration **(**Fig. 1**).**

**Table 3:** Correlation coefficients matrix between obesity indices and bioclinical variables

	Height (cm)	Weight (kg)	Gly (g/l)	TG (g/l)	HDL (g/l)	LDL (g/l)	CRP (mg/l)	SBP (mm Hg)	DBP (mm Hg)
BAI	− 0.53***	0.13**	0.02	0.12**	0.02	0.02	0.06	0.07	0.11**
BFI (kg/m^2^)	− 0.34***	0.32***	0.07	0.14**	− 0.04	0.03	0.08	0.08*	0.11**
WC (cm)	− 0.09*	0.37***	0.13***	0.11**	− 0.15***	0.006	0.11**	0.08*	0.16***
BMI (kg/m2)	0.006	0.80***	0.21***	0.21***	− 2.26***	0.02	0.01	0.19***	0.24***
% total fat mass	− 0.42***	0.006	− 0.01	0.06	0.07	0.02	0.04	0.03	0.02
% trunk fat mass	− 0.22***	0.23***	0.10**	0.15**	− 0.12**	0.01	0.05	0.09*	0.10**

BAI: body adiposity index; BFI: body fat index; WC: waist circumference; BMI: body mass index, Gly: glycaemia, TG: triglycerides, HDL: high density lipoprotein, LDL: low density lipoprotéin, CRP: c-reactive protein, SBP: mean 24 h systolic blood pressure, DBP: mean 24 h diastolic blood pressure

***p < 0.0001; **p < 0.001; *p < 0.01

**Table 4:** Relationship between body indices and metabolic syndrome criteria according to multivariate logistic regression analysis

	Metabolic syndrome criteria number				
Obesity indices	1	2	3	4	5
	OR 95% [CI]				
BAI %	**1.04** [0.97–1.10]	**1.18** [1.19–1.29]***	**1.07** [0.96–1.20]	**1.18** [0.99–1.40]	**1.11** [0.85–1.45]
BFI (kg/m^2^)	**1.09** [0.99–1.20]	**1.22** [1.07–1.40]**	**1.37** [1.14–1.64] *	**1.28** [0.95–1.72]	**1.47** [0.90–2.42]
BMI (kg/m^2^)	**1.27** [0.87–1.84]	**1.33** [0.81–2.18]	**2.94** [1.48–5.85] **	**3.13** [0.98–9.96]	**2.43** [0.37–16.12]
WHR	**1.17** [0.89–1.99]	**1.27** [0.87–2.84]	**1.47** [1.20–1.99] *	**1.69** [0.87–3.84]	**1.57** [0.90–2.85]

BAI: body adiposity index; BFI: body fat index; BMI: body mass index; WHR: waist hip ratio, OR: odds ratio, CI: confidence interval

***p < 0.0001; **p < 0.001; *p < 0.05

**Fig. 2:**
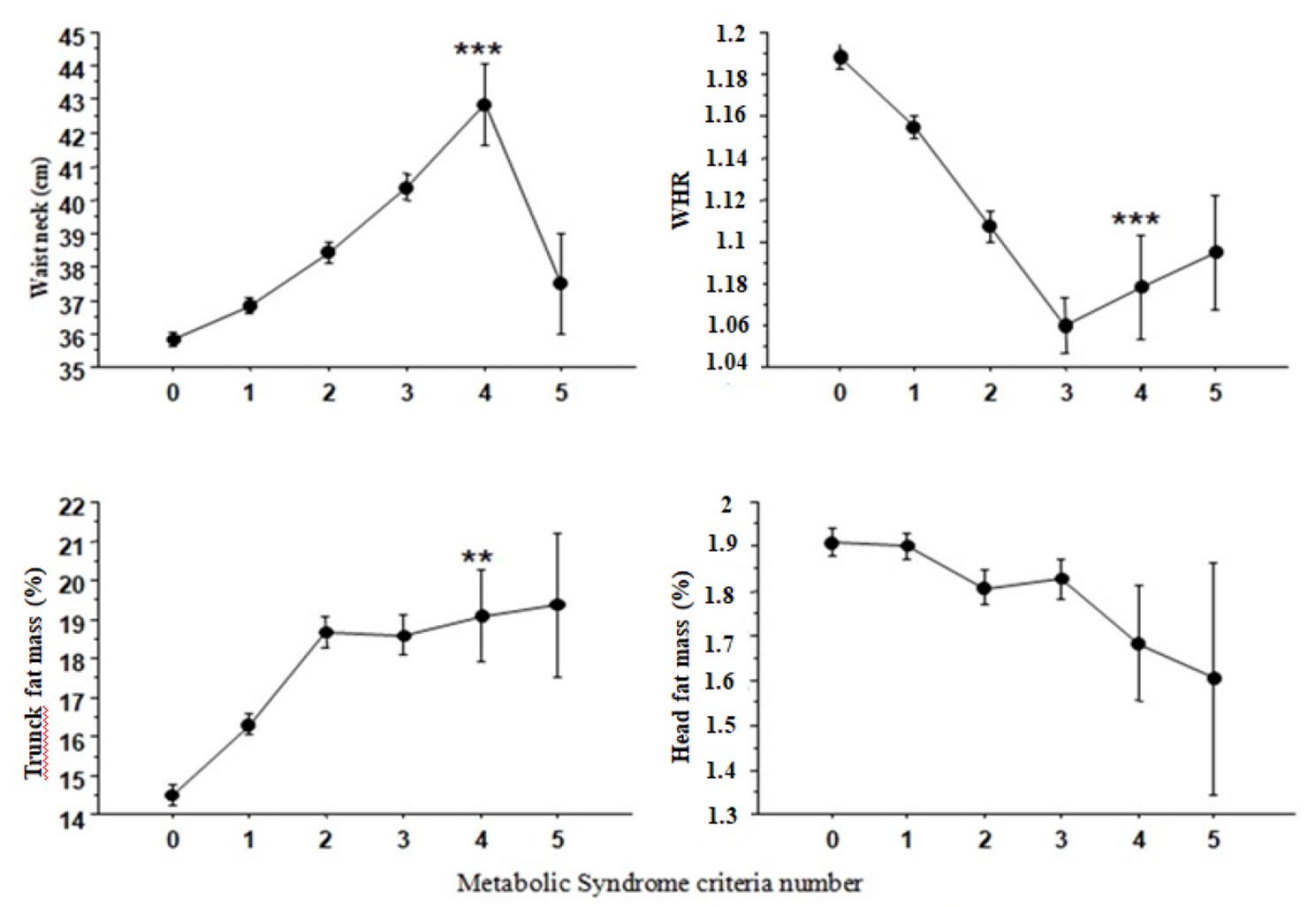
Evolution of physical indices of fat mass according to metabolic syndrome criteria number. WHR: waist to hip ratio ** p < 0.001 ; ***p < 0.0001 (0 vs. 4)

Interestingly, fat repartition was modified with the number of MS criteria, that percentage decreasing for Head fat mass, while increasing for Trunk; the percentage of neck circumference increased then decreased, and the WHR decreased **(**Fig. 2**).** This underlines the central position of the trunk fat mass during the increasing number of criteria.

## Discussion

In this study, Waist to Hip Ratio showed a significant correlation with all bioclinical variables (Table 3), underlining its importance. Conversely, BFI did not correlate with cholesterol, glycemia, and inflammatory markers. BFI and BAI slightly correlated with waist circumference and triglycerides (TG) (Table 3). BAI and BFI better correlate with MS criteria than BMI. For this reason, we can state that BFI and BAI would be two more suitable tools than BMI to characterize obesity [9–11]. Interestingly, all indicators correlate well with diastolic blood pressure, reinforcing its predictive value.

We showed in a previous study that BFI was the best indicator of body fat in relation to the progressive decrease in activity of the autonomic nervous system, a strong predictor of cardiovascular disease [8]. In fact, overall and abdominal obesity as defined according to a BMI > 30 kg/m^2^ and a WC greater than 88 or 102 cm take into account both the lean mass and fat mass. According to previous observations, it seems that BFI remains the best indicator to characterize the composition of body fat, while the BMI seems in relation to the overall weight. All forms of obesity increase the risk of CVD and diabetes [12, 13]. We propose that the markers of a central-distributed obesity, in particular WHR, in this case BAI or BFI in our context, would be more strongly linked to coronary artery disease (CAD) events as compared to BMI when used as a conventional measure (Table 4) [14, 15].

Obesity represents an increasing public health problem all around the world. The incidence and the prevalence of obesity (estimation based on BMI values only) is higher in most developed countries and is the lowest in Asia. Previous studies have explored the relationship or association between BMI, waist-hip ratio or WC and CAD [5, 15]. The results of these studies were contradictory some suggesting that BMI was better, others that BMI is not a quite good marker for abdominal adiposity. Others authors have suggested that markers of abdominal obesity may be better predictive, in subpopulations of younger subjects and in women. Smaller previous studies also reported an opposed relationship between increased hip circumference and diabetes mellitus, systolic/diastolic hypertension, or dyslipidemia, and CVD [15–18]. Our cohort study is a good way of correlating these data as the subjects are relatively good representative of the general European population. However, we understand that choosing NCEP criteria may have given lower figures than IDF or Harmonized criteria.

### Limitations

The inclusion process is a limit of the study, as it is probable that the healthiest people entered the study. However, the cohort was verified as a good sample of the French population [6]. The inclusion was performed by three different doctors, while the populations’ characteristics did not differ between them. The biology was performed by a central laboratory, which decrease the eventuality of biological bias. This manuscript do not present the correlations between the cardiovascular events and the markers presented here. These data are not yet available.

## Conclusions

Among the obesity indices, BFI and BAI represented the best indicators to characterize global obesity. These different indicators are real alternatives for measuring obesity. It should be emphasized that among these cardiometabolic risk assessment parameters, only the BAI can be used in the absence of availability of advanced but cumbersome and expensive techniques such as DXA and many other tomographic techniques. Conversely, the calculation of the BFI requires the prior performance of DXA.

## Data Availability

Due to ethical restrictions approved by the ethics committee of our institution (University Hospital, Saint Etienne, France), the data used in this study can be made available for research proposals by a request to Proof’s Publications Committee.
